# Editorial: Antimicrobial resistance: causes, mechanisms and mitigation strategies for gut dysbiosis

**DOI:** 10.3389/fmicb.2025.1765239

**Published:** 2026-01-12

**Authors:** Avinash V. Karpe, Pooja Hegde, Coralia Bletotu, Zhigang Qiu

**Affiliations:** 1 Commonwealth Scientific and Industrial Research Organisation (CSIRO), Canberra, ACT, Australia; 2 Lilly Research Laboratories, Eli Lilly (United States), Indianapolis, IN, United States; 3 Department of Cellular and Molecular Pathology, Stefan S. Nicolau Institute of Virology, Bucharest, Romania; 4 Tianjin Institute of Environmental and Operational Medicine, Tianjin, China

**Keywords:** antibiotic resistance genes (ARG), conjugative transfer genes, multi drug resistant (MDR), mechanism of action, microbiome and resistome, systems biology

Antimicrobial resistance (AMR) continues to escalate as a major global health challenge, and studies over the last 10 years indicate that gut microbiome—and its expansive resistome—play a key role here. The gastrointestinal tract, a densely populated microbial ecosystem, is both vulnerable to antimicrobial disruption and capable of serving as a dynamic reservoir for resistance genes (Karpe et al., 2023; Ho et al., 2025; Nazir et al., 2025). This Research Topic, *Antimicrobial Resistance: Causes, Mechanisms and Mitigation Strategies for Gut Dysbiosis*, was conceived to address this dual role of the gut: as an ecological victim of antimicrobial pressures and as a driver of the evolution, persistence and dissemination of resistance. The Research Topic brings together diverse contributions that collectively highlight the mechanisms underpinning dysbiosis, the pathways through which resistance emerges and propagates, and the possibilities for novel mitigation strategies.

The Research Topic contains, within it, two mechanistic studies using murine models, each providing detailed insights into how antimicrobial stress shapes gut ecology. In the work by Ding et al., the authors investigate conjugative transfer of antibiotic resistance genes mediated by plasmids across different intestinal segments. Their findings reveal striking anatomical specificity: the small intestine emerges as a primary site for donor strains and plasmid-borne antibiotic resistance genes (ARG) localization. This is accompanied by shifts in microbial richness—rising in the duodenum, jejunum and large intestine, but declining notably in the ileum. The concurrent expansion of Proteobacteria under plasmid-transfer pressure offers further evidence of how mobile genetic elements can reshape intestinal microecology. Complementing this, Ma et al. examined the combined impact of multidrug-resistant *Escherichia coli* and multi-antibiotic exposure. In their study, they observed dramatic reductions in operational taxonomic unit richness, with communities shrinking from over 200 OTUs in controls to just a few dozen in antibiotic-challenged groups. Again, the small intestine was shown to be the key colonization site for resistant strains. Together, these studies expose the profound and segment-specific ecological consequences of antimicrobial and ARG pressures within the gut, pointing to a complex interplay between microbial competition, niche restructuring and gene mobility.

From animal models, the Research Topic shifts to early-life human ecology with the study by Qiu et al. In this study, the authors explored how neonatal antibiotic therapy—specifically amoxicillin–clavulanic acid and moxalactam—affected the initial establishment of the gut microbiota and key functional taxa, notably butyrate-producing bacteria. Their findings of reduced microbial diversity and depletion of butyrate producers in antibiotic-exposed infants highlight how early-life antimicrobial exposure may have enduring consequences. Perturbations during this critical developmental window may influence immune maturation, metabolic programming and the long-term architecture of the gut resistome. This work underscores the importance of stewardship in pediatric settings, where microbiome trajectories are still being established and may be particularly vulnerable to pharmacological disruption.

The Research Topic also includes two comprehensive reviews that provide conceptual frameworks essential for interpreting these experimental findings, and future aspects in building resilience to AMR. In Deshpande et al., the authors frame the gut microbiome as an emerging epicenter of AMR, synthesizing evidence on how reservoirs of resistance genes within commensal communities interact with pathogens via horizontal gene transfer. Their review positions the gut not merely as a site affected by antibiotics but as a crucial engine of resistance evolution. Furthermore, Al-Kuwari et al. presented a perspective pointing toward future translational possibilities: microbiome-based interventions that might complement or even reduce reliance on antibiotics. By exploring strategies such as dietary modulation, probiotics, fermented foods and microbiota restoration, the authors highlight the potential for leveraging microbial diversity to counteract resistance pressures. Their contribution reflects a growing recognition that rebuilding or preserving ecological stability may be as critical as combating individual resistant pathogens.

Finally, the One Health dimension of AMR was presented by Lertwatcharasarakul et al. through their retrospective analysis of antimicrobial resistance in *Salmonella* spp. isolated from livestock and their environment in Thailand. By profiling resistance patterns across animal and environmental samples, they illustrated how antimicrobial use within agricultural and veterinary settings shapes resistance in zoonotic pathogens and their surrounding niches. This work underscores the continuity between gut-associated resistomes in animals and broader environmental reservoirs. Their study also highlighted the risk of bidirectional transmission of resistant organisms between livestock, the food chain and humans. In doing so, it links the gut-centered themes of this Research Topic to wider questions of agricultural practice, environmental stewardship and cross-species transmission.

Altogether, these articles build a coherent narrative: that AMR cannot be solved by pharmaceutical innovation alone, but demands an ecological understanding of the gut microbiome and its role in resistance evolution. The findings reinforce that antimicrobial exposures—whether through plasmid-mediated transfer, resistant organisms or early-life therapies—can dramatically reconfigure gut communities in ways that promote ARG retention and dissemination. At the same time, the reviews and perspectives highlight the promise of resilience-based and microbiome-centric interventions that move beyond conventional antibiotic strategies.

As this Research Topic demonstrates, addressing AMR requires integration across mechanistic biology, microbial ecology, clinical practice and One Health policy. The gut microbiome, long overlooked in AMR frameworks, now stands as a central player. We hope that this Research Topic not only advances understanding of the complex interplay between antimicrobials, gut ecology and resistance, but also inspires future work that blends ecological insight with translational ambition to preserve both microbiome health and antimicrobial effectiveness.

We thank all contributing authors for their high-quality work and all reviewers for their essential input.
